# The Correlation between Chronic Periodontitis and Oral Cancer

**DOI:** 10.1155/2013/262410

**Published:** 2013-07-08

**Authors:** Maximilian Krüger, Torsten Hansen, Adrian Kasaj, Maximilian Moergel

**Affiliations:** ^1^Department of Oral and Maxillofacial Surgery-Plastic Surgery, Johannes Gutenberg University of Mainz, Medical Center, Augustusplatz 2, 55131 Mainz, Germany; ^2^Institute of Pathology, University of Mainz, Medical Surgery, Langenbeckstraße 1, 55131 Mainz, Germany; ^3^Department of Operative Dentistry and Periodontology, Johannes Gutenberg-University, Augustusplatz 2, 55131 Mainz, Germany

## Abstract

Infections are increasingly considered as potential trigger for carcinogenesis apart from risk factors like alcohol and tobacco. The discussion about human papilloma virus (HPV) in oral squamous cell carcinoma (OSCC) points at a general role of infection for the development of oral carcinomas. Furthermore, first studies describe a correlation between chronic periodontitis and OSCC, thus, characterizing chronic inflammation as being a possible trigger for OSCC. In front of this background, we present four well-documented clinical cases. All patients showed a significant anatomical relation between OSCC and clinical signs of chronic periodontitis. The interindividual differences of the clinical findings lead to different theoretical concepts: two with coincidental appearance of OSCC and chronic periodontitis and two with possible de novo development of OSCC triggered by chronic inflammation. We conclude that the activation of different inflammatory cascades by chronic periodontitis negatively affects mucosa and bone. Furthermore, the inflammatory response has the potential to activate carcinogenesis. Apart from a mere coincidental occurrence, two out of four patients give first clinical hints for a model wherein chronic periodontitis represents a potential risk factor for the development of OSCC.

## 1. Introduction

Squamous cell carcinoma is the most frequent malignancy in the oral cavity and with nearly 400.000 new diagnosed patients worldwide each year; it represents the sixth frequent malignant tumor. Despite multimodality approaches for the treatment comprising surgery and adjuvant chemo- and radiation therapy, the disease still has a low overall survival rate of about 50% [1, 2]. The development of new therapeutic strategies with improved treatment options or possible prevention of oral squamous cell carcinoma (OSCC) requests a substantial understanding of its etiology. The last years have revealed more detailed information about different risk factors for the development of OSCC. Important risk factors of the general accepted multistep carcinogenesis model are genetic predisposition [3], presence of premalignant lesions [4], and environmental or behavioural carcinogenic triggers, for example, the ingestion of tobacco and alcohol [5]. Recently, the influence of infection and inflammation for cancer development has been discussed. Associations between human papilloma virus (HPV) infection and oropharyngeal carcinomas have been documented [6]. These patients are typically Caucasians, nonsmokers, nondrinkers, and one decade younger on average than people suffering from HPV negative carcinomas. Intriguingly, patients with HPV-positive oropharyngeal carcinomas had a significant better prognosis than the HPV negative collective [7, 8]. This finding might point at subtypes of infection-induced carcinomas with different clinical behaviours, thus, stressing the need of further characterization. Comparably, the predominant infection within the oral cavity is chronic periodontitis, and its role for the development of oral cancer was likewise recently discussed [9, 10]. Herein, periodontitis occurs as chronic inflammatory process characterized by specific bacteria and the loss of attached gingiva and alveolar bone, with consecutive development of periodontal pockets and loss of teeth [11]. A recently published work by Tezal et al. found the loss of bone as clinical sign for chronic periodontitis being an independent risk factor for the development of carcinoma within the oral cavity [12]. In front of this background, the case series at hand comprises four patients treated at our clinical Department for OSCC. Within these, the synopsis of clinical appearance, radiologic findings, and cross-sectional resection specimen offer an association of the carcinoma to the periodontal space with signs of chronic inflammation. The different clinical aspects are discussed comprising the available literature on this topic.

## 2. Case Presentations

**Case 1**

**Figure 1:**
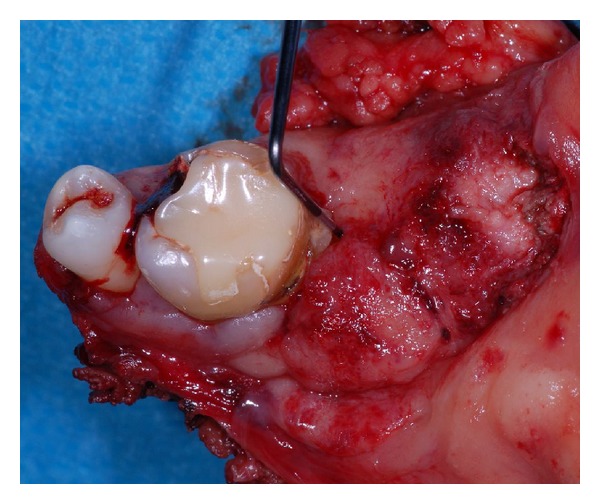
Case  1—resection specimen of the mandible. The probe reveals the loss of clinical attachment within the periodontal space, and the tumor seems to emerge from the periodontal compartment.

**Figure 2:**
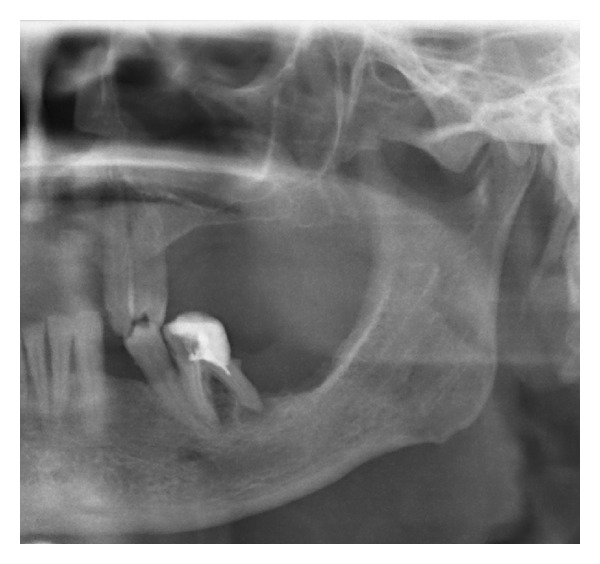
Case  1—the preoperative OPTG exhibits severe horizontal and vertical bone loss of the jaws. In addition, peri- and interradicular osteolytic lesions around tooth 36 are present. A soft radiopacity as projection of the tumor mass can be identified along the intact alveolar crest distal of 36. Radiolucency, suspect for bone invasion is solely present at the interradicular and distal aspect of the roots.

**Figure 3:**
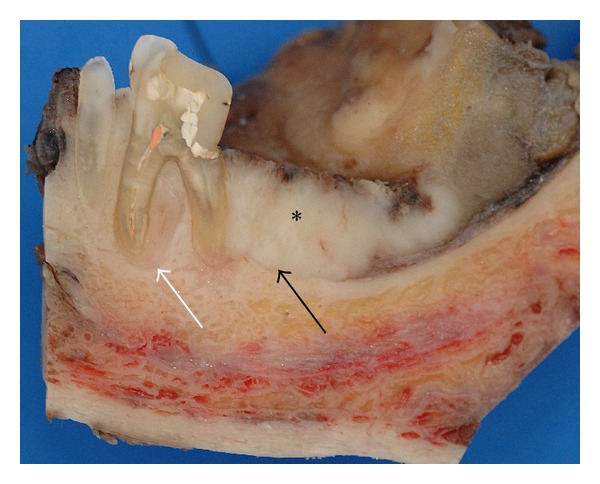
Case  1—cross section specimen. The black arrow highlights the close relation of the distal root to the tumor mass (∗) with preserved cortical bone distal to the tooth. The white arrow points at a tumor formation along the mesial root filling out the interradicular space.

A 59-year-old woman presented herself with an exophytic mass of 2 cm adherent to the mandible and localized distally of tooth 36. The tooth revealed signs of chronic periodontitis with bleeding on probing, attachment loss and a 5 mm deep pocket, and significant mobility on clinical examination. Polymerase chain reaction (Micro-ident, Hain Lifescience GmbH D-72147 Nehren) (PCR) revealed an infection with *Porphyromonas gingivalis* and *Tannerella forsythia* (*red complex*). The expansion of the tumor reached from the sulcus glossoalveolaris with its center along the alveolar crest to the adjacent buccal mucosa (Figure 1). The patient reported herself to be a never smoker and never ingested alcohol. Oral hygiene habits were sufficient. No other precursor lesions like leukoplakia, erythroplakia, or signs of oral lichen planus were found on examination. The visit of our department was induced by progressive pain and increasing swelling. The preoperatively taken orthopantomography (OPTG) revealed a considerable generalized horizontal type of bone loss within the upper and the lower jaw, as also a distinct inter- and periradicular osteolytic lesion at the region of 36 (Figure 2). A both-sided selective neck dissection [13] and partial resection of the mandible followed. The clinical appearance (Figure 1) showed an exophytic mass with contact to the distal root of 36. Intriguingly, the OPTG showed no arrosion of the distal mandible but apparently an inhomogeneous radiolucency between the roots of the molar. The cross-section specimen (Figure 3) furthermore highlights the interradicular spreading of the tumor without an infiltration of cancellous bone or the distal alveolar crest. HPV status by p16 immunostaining was negative.

Taking all clinical findings together, the present case supports the hypothesis of a possible de novo development of malignancy from the interradicular periodontium.

**Case 2**

**Figure 4:**
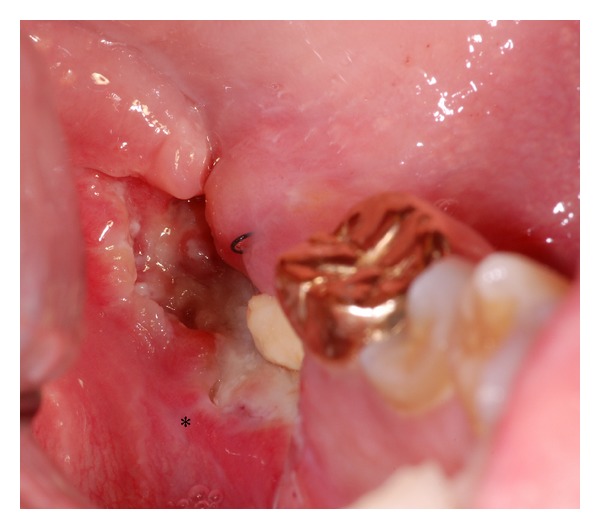
Case  2—the clinical aspect consists of a fibrin-coated ulcer in region of the wisdom tooth at the left lower mandible. The adjacent mucosa shows perifocal signs of inflammation (∗).

**Figure 5:**
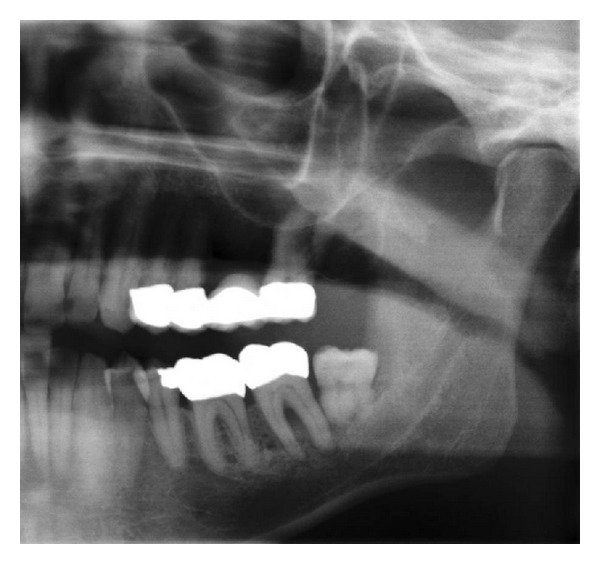
Case  2—Preoperative OPTG—A large formation of enhanced translucency is found in projection of the radices 36 and 37 as also at the pericoronal aspect of the partially retained wisdom tooth.

**Figure 6:**
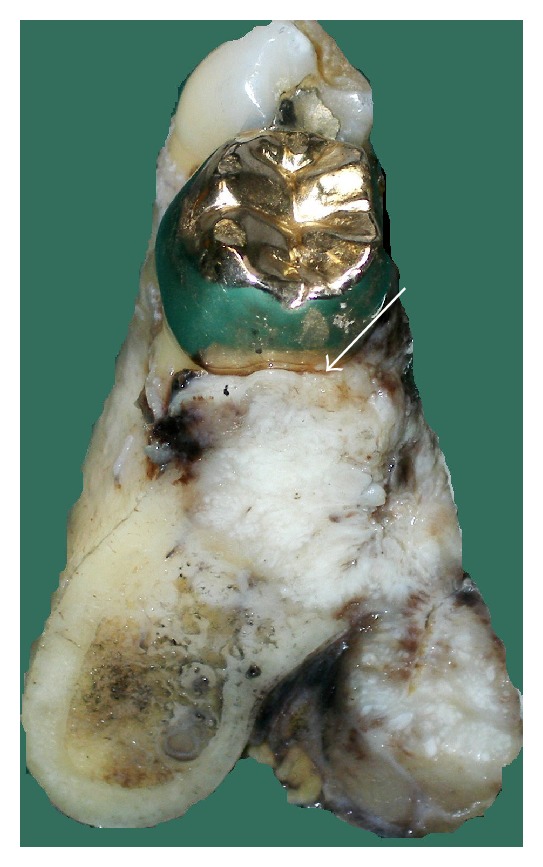
Case  2—cross section specimen with a sectional view from distal after removal of the bony aspect that contained the wisdom tooth. The tumor is in broad contact to the periodontium (arrow) with infiltration of the adjacent cancellous bone and the floor of the mouth.

**Figure 7:**
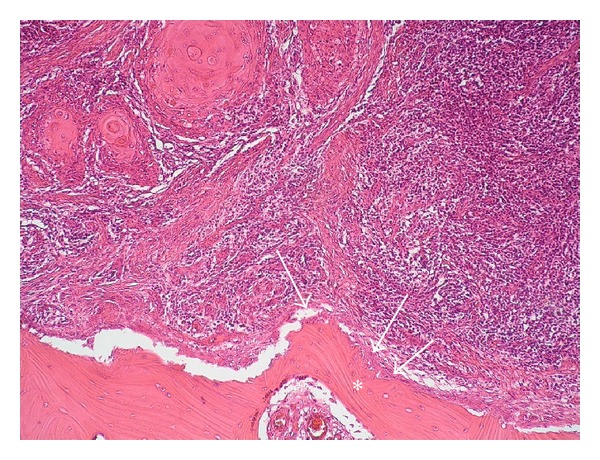
Case  2—histological view of the cross section specimen after Haematoxglin-Eosin staining. The arrows mark the arrosion of the bone (∗) by the tumor cells.

A 48-year-old man presented himself with a histological verified squamous cell carcinoma of the left mandible adjacent to the partially retained tooth 37 surrounded by a bony defect of 3 mm. Analysis of the bacteria (Micro-ident, Hain Lifescience GmbH D-72147 Nehren) inside the pocket showed an infection with *Peptostreptococcus micros* and *Fusobacterium nucleatum* (*orange complex*) as well as *Eikenella corrodens* and *Capnocytophaga* sp. (*green complex*). The patient reported recurring inflammation in the left lower quadrant for a period of six weeks. Prior to the planned osteotomy of the wisdom tooth, the dentist had taken a scalpel biopsy, giving evidence for OSCC. Clinical examination revealed an ulcerous lesion with dominant signs of perifocal inflammation. The ulcer was delineated by a rigid wall of mucosa attached to the alveolar crest related to 37 and 38 (Figure 4). The patient declared to be a nonsmoker but admitted occasional alcohol consumption. Oral hygiene was assessed to be average, and no other precursor lesions were present. The preoperatively taken OPTG (Figure 5) revealed considerable periradicular bone loss in regions 37 and 36 analogous to typical radiologic findings in patients with moderate to advanced chronic periodontitis. The patient underwent radical intended resection of the tumor.  Figure 6 shows the resection specimen from a distal view. The tumor is mainly localized in the interdental space of 37 and 36 with distinct relation to the periodontal space of the crown 36. The beginning infiltration of the mouth floor becomes apparent.  Figure 7 shows the microscopic view with arrosion of the bone by the tumor cells. HPV status by p16 immunostaining was negative.

**Case 3**

**Figure 8:**
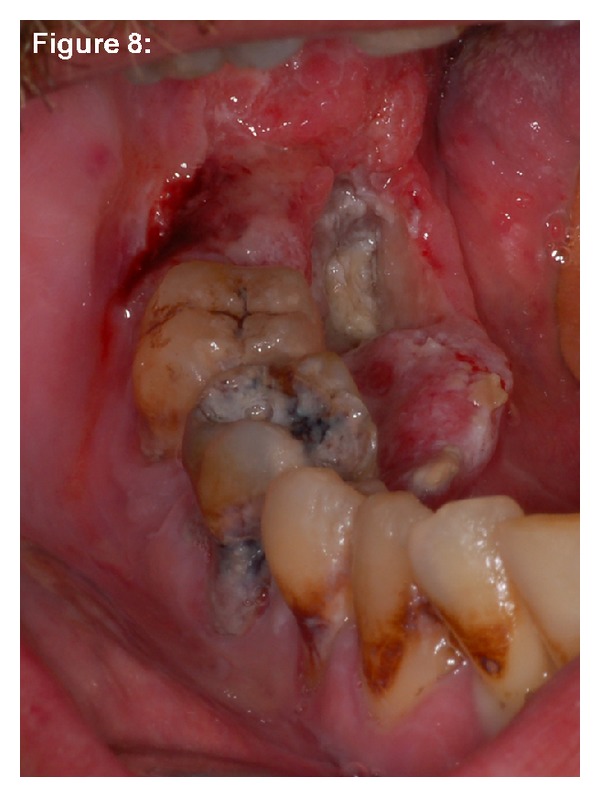
Case  3—clinical situation. A partially ulcerous tumour formation covered the lingual aspect of the alveolar crest adjacent to the two distal molars (46 and 47). Nearly all teeth of this quadrant show clearly the sequelae of nearly nonexistent oral hygiene habits. Striae of leukoplakia are found on the wall of the ulcer, the tongue, and also along the vestibular papillae.

**Figure 9:**
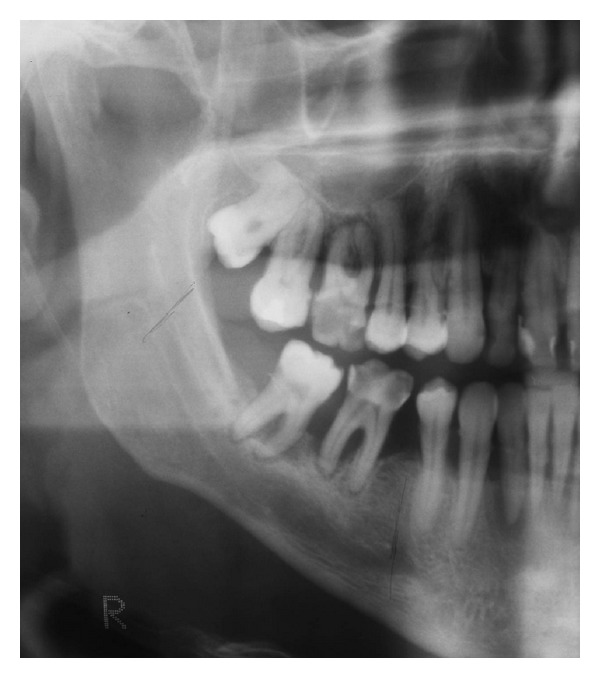
Case  3—preoperative OPTG. Beside multiple carious lesions the present X-ray examination reveals signs of chronic periodontal disease with a general loss of horizontal bone level, liberation of both dental roots, and bifurcations. The local maximum of destruction is found in the region of the last two molars. The retromolar triangle, however, appears to be intact.

**Figure 10:**
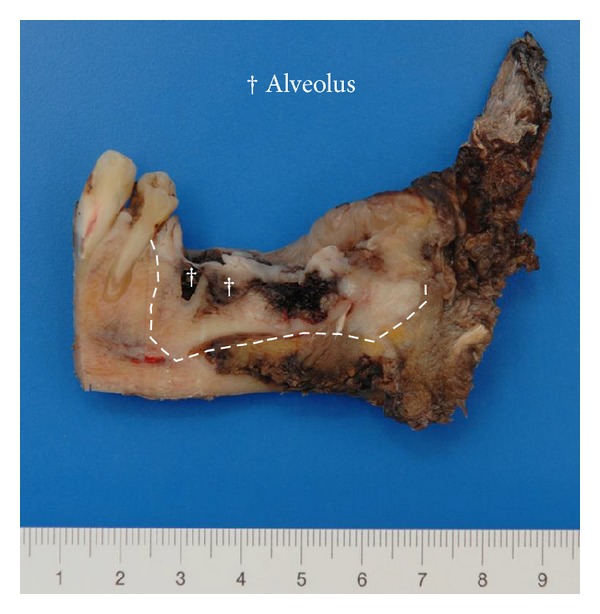
Case  3—the sagittal split preparation of the resection specimen from a lingual view clearly shows the association of the tumor (borders marked with lines) to the dental alveoli (†) of the distal molars. The teeth 36 and 37 are lost due to the preparation process.

A 50-year-old man was referred to our clinic by a maxillofacial surgeon with diagnosis of a squamous cell carcinoma of the right mandible. The clinical examination revealed a tumor of 4 cm in diameter, localized at the alveolar crest distal of 46 with extensions to the adjacent floor of the mouth. Around the ulcer, fields of homogeneous leukoplakia were detectable (Figure 8). The patient suffered from generalized advanced chronic periodontitis, exhibiting pocket depths up to 7 mm with bleeding on probing. Analysis of bacteria (Micro-ident, Hain Lifescience GmbH D-72147 Nehren) revealed infection with *Porphyromonas gingivalis*, *Tannerella forsythia*, *Treponema denticola* (*red complex*), *Peptostreptococcus micros*, *Fusobacterium nucleatum* (*orange complex*), and *Campylobacter rectus* (*orange*-*associated complex*). Oral hygiene was extremely poor with high amounts of soft and hard debris on the furthermore carious teeth. The patient reported to be a heavy smoker (60 pack years) and drinker (>3 L beer a day). The OPTG (Figure 9) exhibited severe general bone loss with a basin-like translucency comprising the last distal molars of the right mandible. A radical intended operation followed.  Figure 10 shows a transverse section of the resected mandible from a lingual view. Herein, the tumor surrounds the empty alveoli of the artificially removed right molars with considerable infiltration of the alveolar bone. HPV status by p16 immunostaining was negative.

**Case 4**

**Figure 11:**
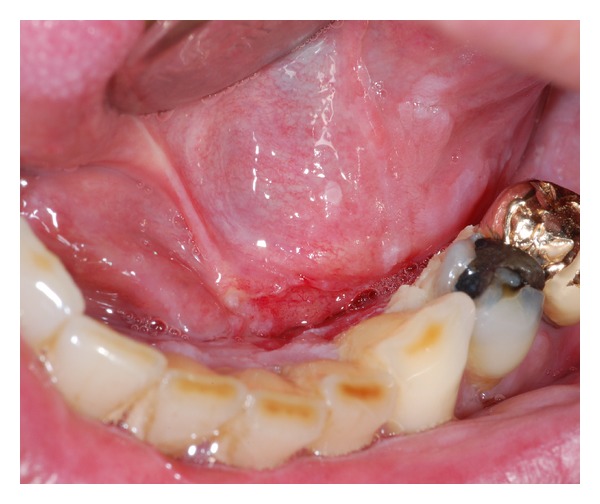
Case  4—a superficial ulcerous lesion atop of a mass at the left sublingual space with extension to the lingual aspect of the alveolar crest comprising the teeth 31 to 34 and surrounding leukoplakia.

**Figure 12:**
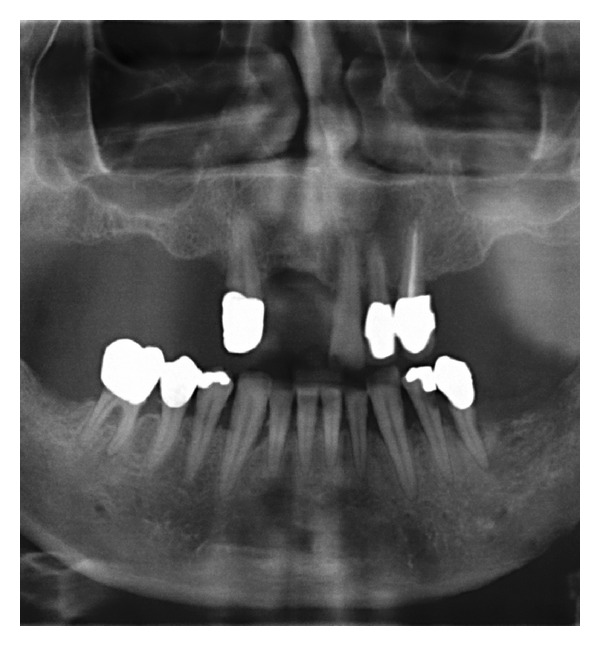
Case  4—preoperative OPTG. A general horizontal loss of bone in both jaws with additional vertical translucencies in the front aspect of the mandible and signs of erosion of the alveolar crest at the left lower quadrant.

**Figure 13:**
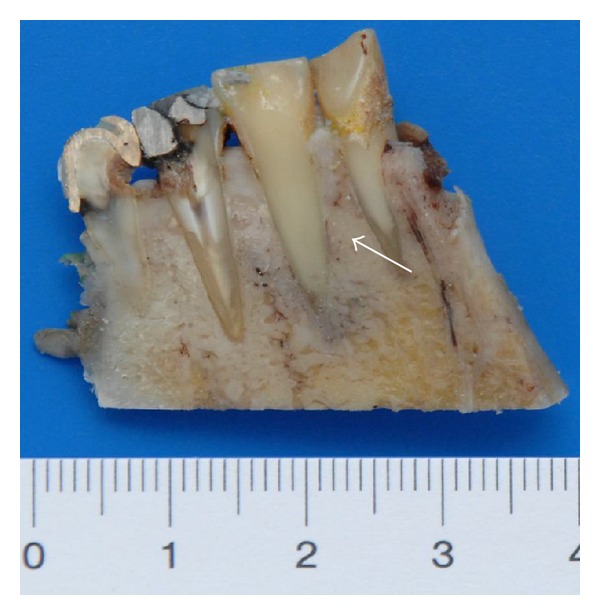
Case  4—the split resection specimen from a lingual view demonstrates the association of tumor formation along the root of the left lower canine (white arrows) as also further invasion of the cancellous bone.

**Figure 14:**
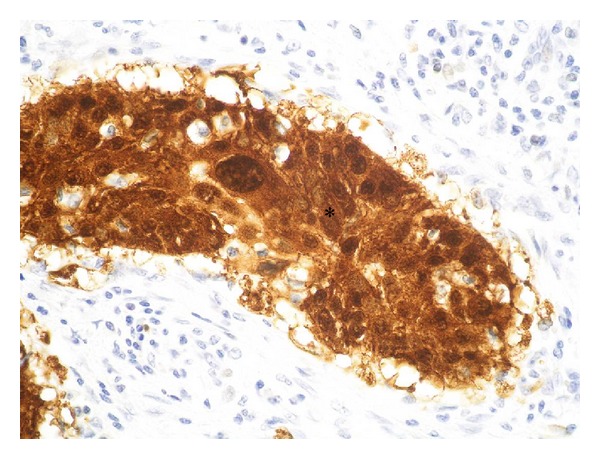
Case  4—histological view of the tumor after immune-histochemical staining against p16. The brown signal (∗) proves the infection with HPV.

A 53-year-old man was admitted to our clinic with the histological verified diagnosis of a squamous cell carcinoma of the anterior floor of the mouth. The patient noticed an indolent growing mass five weeks before admission.  Figure 11 gives a picture of the clinical situation. Beside a space consuming sublingual mass with deviation of the lingual frenulum, a small erosive lesion lingual from the left lower anterior teeth (region: teeth 31 to 34) with subtle perifocal leukoplakia around the center of the lesion is shown. The examination revealed a solid tumor formation with its center mainly on the left floor of mouth. The tumor expanded from the left angle of the mandible with a midline crossing to the region of the first right lower incisor. Adherence to the bone was detectable in the region of the left lower canine and the first premolar. The oral hygiene status was moderate. Investigation of periodontal pockets revealed depths up to 4 mm and bleeding on probing. The patient smoked 10 cigarettes per day for ten years and occasionally drank a glass of wine. The OPTG (Figure 12) revealed a generalized horizontal type of bone loss within both jaws, with additional vertical bony defects and translucency as radiologic finding for an erosive process in the left anterior lower jaw. The radical intended surgery consisted of a bloc resection including parts of the mandible, tongue, floor of mouth, and hyoid with complete bilateral selective functional neck dissection [13]. The split-resection specimen (Figure 13) shows a periradicular tumor formation along the root of the canine with infiltration of the neighboring cancellous bone. HPV status by p16 immunostaining was positive, as shown in Figure 14.

A brief summary of all cases is given in Table 1.

## 3. Discussion

Our study presents four thoroughly documented cases with alveolar squamous cell carcinomas being directly associated to teeth that show considerable signs of chronic periodontitis. Chronic periodontitis represents the most common infection worldwide with high clinical relevance for the dentist. In front of this clinical setting, four different models of chronic periodontitis with alveolar squamous cell carcinomas are discussed as follows. The first patient had no precursor lesions, and no other risk factors had been identified except clinical signs of chronic periodontitis. In this patient, a de novo development caused by genomic instability as consequence of chronic inflammation caused by gram-negative bacteria as postulated by Guerra et al. appears possible [14]. In this model, chronic periodontitis itself would trigger the development of oral squamous cell carcinoma. Recently, there is improving interest in the development of various types of cancer and their association to inflammation as also the underlying pathophysiological mechanisms that lead to malignant transformation [15]. One important factor in cancer-related inflammation is the transcription factor NF-*κ*B. Beside its function as key coordinator of innate inflammation and immunity by activated expression of inflammatory cytokines, adhesion molecules, and angiogenic factors, it has also been identified as endogenous tumour promoter [16]. Moreover, it is substantially involved in the inflammatory process of chronic periodontitis [17]. In addition, an association between oral cancer and chronic mechanical trauma was also described [18], suggesting that inflammation independent of its cause may predispose to cancer. Hereby, malignant transformation of oral epithelium would be a consequence of the immune response like macrophage and T-cell activation and cytokine release (e.g., IL-1, IL-8, and TNF-*α*) [19]. Aside malignant transformation as sequel of unspecific inflammation, a specific bacterial, or viral agent may also promote malignancies. This sequence is supposed for gastric lymphomas [20], *H. pylori* infection and gastric cancer [21], Hepatitis B Virus (HBV) and HCV infection in liver cancer [22] and HPV 16/18 infection in head and neck [23] or cervical cancer [24]. Herein, microbial activation of inflammatory cells leads to a respiratory burst and release of free radicals, which can contribute transformation to malignancy by DNA damage, peroxidation of lipids, or disturbance of physiological posttranslational modification of proteins [25]. Taken together, either genomic instability directly induced by the bacterial agent itself or as consequence of immunological response to chronic inflammation, both are main characteristics of chronic periodontitis. The clinical relevance of chronic periodontitis for the development of OSCC was investigated by Tezal et al. In a case control model, the loss of bone as clinical sign for chronic periodontitis was an independent risk factor for tongue carcinomas and was still of significance in a multiple regression model [12]. Particularly, these patients would benefit from periodontal therapy in terms of primary prevention. The second case offers another possible scenario. Here, chronic periodontitis acts as promoter for the invasion of tumor cells into the bone. During the course of chronic periodontitis, the loss of clinical attachment level and the underlying bone is substantially triggered. The periodontal-localized inflammation macerates the cancellous bone by enhanced osteoclastic activity which may constitute a potential route for invasion of an adjacent carcinoma. Osteoclastic activity is enhanced by proinflammatory molecules like IL-1 or LPS [26], which can be found in gingival crevicular fluid (GCF) during chronic periodontitis [27]. Furthermore, seven potential routes for invasion of the mandible by OSCC have been reviewed by Brown [28]. Beside the occlusal route, neural foramina, attached gingiva, cortical bone defects in the edentulous ridge, or infiltration by secondary tumors in the neck through the lower border, the periodontal membrane in the dentate mandible was hypothesized as a possible way for tumor invasion by Bhattathiri and Nair in 1991 [29]. Supposing the periodontal space a weak point for tumor invasion, the instance of periodontal inflammation may promote an invasion of the mandible by a neighbored tumor [15, 19]. The cross- sectional specimen of the second case underlines this possible scenario with a tumor infiltrating the periodontal space, the surrounding adjacent cancellous bone, and the neighbored floor of the mouth.

The third case is an example for chronic periodontitis being a well-known comorbidity in oral cancer patients. The patient was a heavy smoker and drinker with insufficient oral hygiene. All these habits are for themselves potential risk factors for the development of oral cancer and chronic periodontitis [5, 30–32]. In this patient, chronic periodontitis and OSCC seem to be a coincidence without an option to uncover cause and effect.

An accredited model for the development of OSCC is the multistep theory [33]. Herein, the oral mucosa undergoes different developmental stages from hyperkeratosis over different degrees of dysplasia to invasive cancer, while each level shows consecutive accompanying alterations within the genetic profile [34]. Accordingly, it appears possible, that chronic periodontitis may trigger the pathogenesis of precancerous lesions. In case four, the clinical inspection of the oral cavity revealed a vast field of leukoplakia at the anterior floor of mouth. Besides, the clinical manifest OSCC and multiple teeth were affected by chronic periodontitis. A cross-interaction between the special inflammatory milieu with enhanced levels of proinflammatory cytokines and a change in the bacterial environment may induce the development and progression of precancerous lesions in the alveolar mucosa. This thesis was reinforced recently by Meisel et al., who identified chronic periodontitis as a risk factor for the development of leukoplakia predisposing for oral cancer [35].

## 4. Conclusion

We presented four clinical scenarios of OSCC in the neighbourhood of teeth affected by chronic periodontitis for discussion of the clinical relevance. Clinical experience characterizes most OSCC as coincidence since chronic periodontitis and the oral malignancy share multiple risk factors impeding a definition of cause and effect. On the other hand, we found clinical hints for an interaction of OSCC and chronic periodontitis by means of invasion route preformation or a promotive effect on present precursor lesions. Finally, we give an example for a possible de novo synthesis of the OSCC in a patient without other risk factors. These patients, in particular, would greatly benefit from early therapy of the underlying chronic periodontitis in terms of a primary prevention.

## Figures and Tables

**Table 1:** Summary of the presented cases.

Patient	1	2	3	4
Gender	Female	Male	Male	Male
Age	59	48	50	53
Smoking	Never	Never	60 py	5 py
Alcohol	Never	Occasionally	Regularly	Occasionally
Oral hygiene	Sufficient	Sufficient	Worse	Moderate
Tumor localization	36	37	46	32
Radiologic signs of periodontal disease	Yes	Yes	Yes	Yes
Probing depths	5 mm	2 mm	7 mm	5 mm
TNM classification	pT2 pN2b pMx G2 R0	pT4 pN0 pM0 G2 R1	pT4a pN2b pM0 G2 pR0	pT4a pN2c pM0 G3 R2
Bleeding upon probing	Yes	No	Yes	Yes
Periodontal marker bacteria	Red complex	Orange and green complex	Orange and orange-associated complex	Not identified
HPV status (p16)	Negative	Negative	Negative	Positive
